# Good nutrition across the lifespan is foundational for healthy aging and sustainable development

**DOI:** 10.3389/fnut.2022.1113060

**Published:** 2023-01-24

**Authors:** Leocadio Rodríguez-Mañas, Robert Murray, Carole Glencorse, Suela Sulo

**Affiliations:** ^1^Service of Geriatrics, Getafe University Hospital and CIBER on Frailty and Healthy Aging (CIBERFES), Getafe, Spain; ^2^Department of Pediatrics, Emeritus, The Ohio State University College of Medicine, Columbus, OH, United States; ^3^Abbott Laboratories Ltd., Maidenhead, United Kingdom; ^4^Abbott Laboratories, Abbott Park, IL, United States

**Keywords:** nutrition, malnutrition, lifespan, healthy aging, wellbeing

## Abstract

Ensuring healthy lives and promoting wellbeing across the age spectrum are essential to sustainable development. Nutrition is at the heart of the World Health Organization (WHO) Sustainable Development Goals, particularly for Sustainable Development Goal 2/Subgoal 2, which is to *End all forms of malnutrition by 2030*. This subgoal addresses people of all ages, including targeted groups like young children and older adults. In recent decades, there have been marked advances in the tools and methods used to screen for risk of malnutrition and to conduct nutritional assessments. There have also been innovations in nutritional interventions and outcome measures related to malnutrition. What has been less common is research on how nutritional interventions can impact healthy aging. Our *Perspective* article thus takes a life-course approach to consider what is needed to address risk of malnutrition and why, and to examine how good nutrition across the lifespan can contribute to healthy aging. We discuss broad-ranging yet interdependent ways to improve nutritional status worldwide—development of nutritional programs and policies, incorporation of the best nutrition-care tools and methods into practice, provision of professional training for quality nutritional care, and monitoring health and economic benefits of such changes. Taken together, our *Perspective* aims to (i) identify current challenges to meeting these ideals of nutritional care, and to (ii) discover enabling strategies for the improvement of nutrition care across the lifespan. In harmony with the WHO goal of sustainable development, we underscore roles of nutrition to foster healthy human development and healthy aging worldwide.

## 1. Background

As the world population grows and longevity increases in the 21st century, the need for sustainable development challenges us to ensure health and wellbeing across the age spectrum. In 2015, the United Nations (UN) established 17 *Sustainable Development Goals* (SDG), which were adopted by all Member States. With a target of achievement by 2030, the UN seeks a better future for all people (1). Two SDG are fundamental and interrelated: *Zero Hunger* (SDG #2) and *Good Health and Wellbeing* (SDG #3) (1). The World Health Organization (WHO) has a complementary initiative on healthy aging, with 2020–2030 being recognized as the Decade for Healthy Aging (2–4). Aging well includes components of physical, mental, social, and emotional wellbeing—all of which can affect or be affected by dietary intake and nutritional status (1, 5–8). Indeed, nutrition is at the heart of sustainable development; meeting nutritional needs from infancy through old age builds a framework for health and wellbeing throughout life.

A specific subgoal for SDG #2 (subgoal 2) is to end all forms of malnutrition by 2030, particularly among targeted groups like young children and older adults. In recent decades, we have seen advances in the tools and methods to screen for risk of malnutrition and to assess nutritional status. As well, we have seen improvements in nutritional intervention strategies, treatment products, and methods for measuring treatment outcomes. Nevertheless, research is lacking on *how* nutritional interventions impact healthy aging. This *Perspective* article thus takes a life-course approach to define *what* is needed to address malnutrition and *why*, and to examine *how* good nutrition across the lifespan contributes to healthy aging. We posit that improvement of global nutrition requires broad-ranging approaches—developing nutrition programs and policies, incorporating the best nutrition-care tools and methods into practice, training professionals for quality nutritional care, and monitoring the health and economic benefits of such changes. Here we discuss the following strategies, which are summarized in Supplementary Box 1:

• A focus on updating nutritional policies can help move food systems toward healthy and sustainable food production by leading the way to recognizing healthy and sustainable diets (9). For population health, policies can build food sufficiency for people of all ages worldwide. For individuals, policies can promote intake of healthy foods (e.g., fruits and vegetables, whole grains, legumes, nuts, virgin olive oil (10), and seeds) and reduced consumption of unhealthy foods (e.g., processed red meats, sugary snacks) (11).
• It is important to identify and incorporate best measures of nutritional status into clinical practice—tools for screening, assessment, and outcome measures of nutrition-related growth and health (12). Such methods can flag healthy and unhealthy aging (13, 14). Various measures identify individuals who need nutritional intervention, and they reflect post-intervention health outcomes for individuals and populations.
• To integrate new methods and tools, an early practical challenge is to achieve consensus among health professionals on use of age-specific tools, methods, and indicators of nutritional status for all segments of the population (15–22). Based on such evidence, healthcare professionals then need to develop expert guidelines on use of these tools and measures, and to identify age- and population-specific cutoffs for measures of nutritional and functional status. Further, health professionals need clinical education and training programs on best-practice nutritional care for people of all ages (23–26).
• As new nutritional policies, programs, and methods are incorporated into practice, it is essential to monitor effectiveness of such changes in achieving sufficient and healthy dietary intake in cost-effective ways (27–30). We ask the question, “*What is the value of nutrition*?”

This *Perspective* aims to (i) identify current challenges to end malnutrition and deliver high quality nutritional care, and to (ii) discover enabling strategies that can enhance nutritional care and can support sustained health and wellbeing across the lifespan.

## 2. Nutrition matters across the lifespan

**FIGURE 1:**
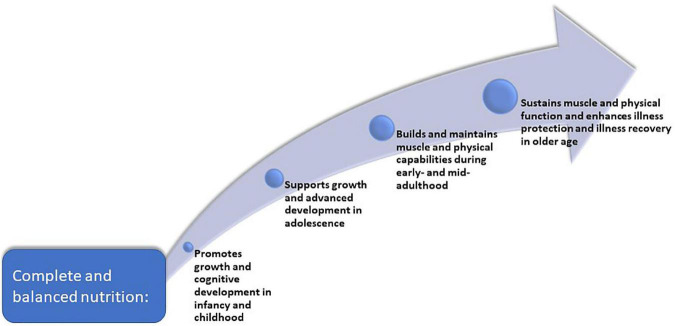
Nutrition across the lifespan is foundational for health and wellbeing in older age.

In the WHO model, healthy aging is viewed as a life-course progression (31, 32). Healthy nutrition is foundational for supporting physical growth and mental development in infants, young children, and adolescents; in young adulthood and midlife, nutrition and lifestyle patterns influence accumulation and maintenance of muscle, thus supporting aspects of health and physical function (Figure 1). In early life, nutrition helps build reserves for mental and physical function (31–34). In later adulthood, age-related decline is expected, but individuals with healthier growth at the beginning of life build biological reserves that prevent or delay age-related disabilities later (14, 35). In older age, good nutrition, including supplemental nutrition when needed, can be used to prevent functional decline and to restore health following acute or chronic diseases, injury, or surgery (36). Together, achieving good nutrition from youth through mid-adulthood is particularly relevant to muscle strength and functionality in older age; goals are to build peak muscle in youth, maintain muscle strength and function in midlife, and sustain muscle function or minimize loss in older age (Supplementary Figure 1) (32, 37).

### 2.1. Grow well to age well: Infancy, childhood, and adolescence

Optimal nutrition is critical from infancy through adolescence and puberty to support growth and to achieve important motor, language, and social milestones (34, 38). Nutritional requirements for children in the first years of life support rapid linear growth and weight gain for increased bone length and muscle mass and for growth and development of the gastrointestinal tract, cardiorespiratory system, kidneys, and immune and central nervous systems (39). Brain development is especially rapid in the first 24 months, thus requiring adequate macro- and micronutrients (38, 39). Without good early nutrition, a child may experience diminished cognitive performance throughout life (39). In middle childhood (5–9 years) and early adolescence (10–14 years), healthy children experience steady linear growth and development, which are supported by adequate nutrition relative to body size (34). During puberty, adolescents (15–19 years) grow and develop rapidly, which requires higher amounts of energy, protein, and micronutrients (34). For lifelong functionality, wellbeing, and achievement, adequate intake of energy, protein, and micronutrients in youth is thus key to physical growth, along with motor, language, and socioemotional development (34, 38–40).

### 2.2. To age well, stay well: Early adulthood and midlife

Staying well in early adulthood and midlife further builds a foundation for healthy aging. Yet some chronic diseases are now increasingly common among younger-aged adults and adolescents, often because of poor nutrition, lack of physical activity, and smoking (41). The WHO estimates that the elimination of these behaviors would decrease the risk of cardiovascular disease, stroke, and type 2 diabetes by 80% in older adults (41). Focus on nutrition, weight management, and physical activity in the middle years can facilitate longer and healthier lives.

In terms of nutrition, healthy aging is based on cumulative effects during the early and middle years of adult life (33). Food insecurity and unhealthy diets are problems that can affect low-, middle-, and high-income countries, although underlying causes differ as demographic, political, or economic conditions vary (11). Notably, middle-aged adults in the United States (US) consume less than the recommended daily requirements for most essential micronutrients leading to deficiencies that increase risk for certain chronic conditions (33). The prevention of osteoporosis in later life, for example, depends on the intake of calcium during childhood and young adulthood to achieve peak bone mineral density by age 30 years (33). Other examples include evidence supporting long-term use of dietary flavanol intake for reducing risk of later Alzheimer’s disease and dementia (42) and long-term adherence to the Mediterranean diet, which can lead to significant reductions in frailty, cardiovascular complications, and macular degeneration in older adults (33). Taking dietary supplements such as vitamin D, calcium, B12, and other micronutrients during the middle years, along with a healthy diet, can help prevent age-associated deficiencies and adverse health consequences (33, 43).

#### 2.2.1. The importance of weight management

Weight management for maintenance of a healthy body mass index (between 18.5 and 24.9 kg/m^2^) (44) plays a key role in midlife and helps decrease risk of developing age-related conditions. The WHO recently reported that 1.9 billion adults worldwide are overweight or obese, while 462 million are underweight (45). Undernutrition and underweight in adulthood, often associated with poverty or illness, lead to conditions such as frailty, osteoarthritis, and impaired mobility, while overweight and obesity increase risk of sleep apnea and urinary incontinence, as well as type 2 diabetes, cardiovascular disease, and cancer (33). Thus, sufficient and healthy dietary intake (fruits, vegetables, whole grains, legumes, nuts, seeds, seafood, healthy oils, and moderate amounts of carbohydrates) is important to achieving weight goals and long-term health benefits (10, 33, 43).

#### 2.2.2. Healthy nutrition and physical activity

A lifestyle of healthy nutrition and physical activity in early adulthood and midlife is key to aging well. Healthcare professionals can incorporate routine nutrition screening into clinical and office-based care for all adults. While there is currently not a well-validated nutritional screening tool for free-living adults in midlife, tools for hospitalized patients can be adapted for community use (33). In addition, the new Remote Malnutrition APP test (R-MAPP) tool was recently introduced for remote screening of adults for nutritional risk and for loss of muscle mass and function when healthcare access was limited during the COVID-19 pandemic; R-MAPP identifies risk for malnutrition and sarcopenia by combining Malnutrition Universal Screening (MUST) and Strength, Assistance in walking, Rise from a chair, Climb stairs, and Falls (SARC-F) tools (18, 22). Advice on maintaining physical activity during midlife and into older age further supports healthy aging, preserves musculoskeletal function, and helps lessen effects of decreased energy intake with aging (33, 46).

### 2.3. To age well, eat well: Older people

The global population of older people (≥ 60 years) is predicted to double over the next three decades—reaching 2.1 billion by 2050 (2). Strategies and systems are thus needed to ensure aging well in this latter phase of life to improve their quality of life and to limit potentially outsized increases in healthcare burdens and costs (2). To this end, health professionals propose a shift from disease-centered care to function-centered care for older people (47, 48). In fact, older people themselves want to maintain function (both physical and mental) (5), an aim reflected in the WHO’s goals for facilitating *healthy aging* (2–4, 49, 50). WHO now frames this concept as intrinsic capacity, which refers to the combination of physical and mental abilities (3). Thus, *healthy aging* refers to sustained intrinsic capacity, while lowered intrinsic capacity reflects functional decline. There is agreement that intrinsic capacity has multiple dimensions—including locomotion, vitality, sensory, cognition, and psychological domains. However, the methods for assessing each of these five dimensions presently differ across studies (51).

Among older people, proper nutrition is a key strategy to prevent or delay onset of chronic disease and functional decline, and to support quality of life—that is, to support *aging well* (52, 53). Conversely, malnutrition (deficient or excess intake of macronutrients or deficient intake of specific micronutrients) can lead to loss of intrinsic capacity with declining physical and mental health (54–57). In fact, there is substantial evidence that poor nutrition is a mediator of adverse outcomes in older people, e.g., lower physical function (58), poorer quality of life (59), greater risk for development of sarcopenia and frailty (53, 60, 61), cognitive decline (57), and shorter survival (62, 63).

#### 2.3.1. What strategies can healthcare professionals use to identify and treat malnutrition and its risk in older people?

Healthcare experts worldwide have developed nutritional guidelines for general health and for muscle health in older people (14, 43, 64, 65). Guidance from the United States of America (USA) advises older adults to improve dietary intake by increasing consumption of fruit, vegetables, whole grains, and dairy, while ensuring that protein intake meets recommendations (43). European protein-intake recommendations have been set higher for older adults compared to those for younger ages, as there is evidence that older adults’ protein requirements are increased to maintain functionality (66, 67). Limited intake of added sugars, saturated fat, and sodium is also recommended for older adults to help manage or avoid chronic conditions (43). A recent study in Spain identified nutrient deficiencies in frail older adults, specifically protein, ω-3 fatty acids, retinol, ascorbic acid, niacin equivalents, folic acid, magnesium, and potassium (68). Other studies have implicated adequate intake of antioxidant and anti-inflammatory micronutrients (vitamins of the B group and vitamins A, C, D, and E) as important to maintaining cognitive health (57). Asian guidelines highlight additional practices to reduce risk of malnutrition and sarcopenia, i.e., (i) offering older adults nutritional counseling on good dietary patterns, and (ii) providing oral nutritional supplements (ONS) when indicated (14).

For clinical and public health professionals, steps to improved nutrition in older people are like those for younger people—*screen* routinely for nutritional risk, *assess* patients at risk to diagnose malnutrition severity, and *intervene* with nutritional care when needed based on a personalized nutrition plan (69). Continued monitoring is important to determine whether the selected nutritional intervention plan is effective. Numerous nutritional screening and assessment tools can be used for older people who are hospitalized (70, 71) or living in the community (15, 72).

Tools have been developed and validated specifically for screening older people and have been shown to predict morbidity and mortality (8, 16, 73). For use in the community, there are several nutritional screening, assessment, and diagnosis tools that can be used; Mini Nutritional Assessment short form (MNA-SF; a validated 6-question tool to rapidly assess geriatric patients for malnutrition or its risk) and Global Leadership Initiative on Malnutrition (GLIM; a tool that assesses three phenotypic [weight loss, low body mass index, and low skeletal muscle mass] and two etiologic [low food intake and presence of disease with systemic inflammation] criteria, with diagnosis confirmed by any combination of one phenotypic and one etiologic criterion fulfilled) criteria are commonly used (73–75).

Patients recognized as malnourished or at malnutrition risk need a more detailed nutritional assessment to identify and quantify specific nutritional problems. Such an assessment includes subjective and objective parameters—medical history, current and past dietary intake (including energy and protein balance), physical examination and anthropometric measurements, functional and mental assessment, quality of life, medications, and laboratory values (69).

When a patient’s nutritional assessment shows evidence of nutritional risk, malnutrition, sarcopenia, or frailty, a diagnosis can be made, and a healthcare professional must develop a personalized nutritional care plan. Implementation of nutritional intervention is particularly urgent for those who are already malnourished or have muscle impairment (41, 76, 77). A Canadian initiative proved effective for avoiding development of frailty (78), while results of a recent European study demonstrated that nutritional care could prevent loss of mobility (79).

#### 2.3.2. How can healthcare professionals monitor the effectiveness of interventional strategies?

The WHO’s Integrated Care for Older People (ICOPE) recommendations advise (i) measuring dimensions of intrinsic capacity to quantify the severity of malnutrition-associated functional decline, and (ii) monitoring these dimensions during and after nutritional interventions (3). Recommendations for managing declines in functionality/intrinsic capacity include dietary advice and use of oral nutritional supplementation. For individuals’ physical health, measures of physical function, ability to perform daily life activities, and strength are used (3). For their mental health, perceived quality of life, cognition, and psychological wellness can be measured (3). In addition to nutritional care, individuals can benefit from exercise or physical activity programs, cognitive care, and encouragement of social connections (79–82). For healthcare systems, nutritional interventions for care of older people can also be measured in terms of resource use, cost savings, and cost-effectiveness. Supplementary Figure 2 provides a summary of strategies to quantify nutrition-related health outcomes in older adults.

#### 2.3.3. What does the recent evidence show about nutritional interventions and health outcomes for older people?

People in the older population, including those living in the community, are among the most vulnerable to malnutrition. Poor nutrition in older people commonly leads to declining physical function, along with social and economic burdens (58, 83–88). However, these problems have often gone unrecognized until very recently. Here we present a detailed summary of recent reports from studies of older people around the world. We summarize up-to-date information and evidence on nutrition screening and assessment, nutritional intervention and outcomes, and implications for nutritional care in clinical practice (Table 1), thus providing evidence-based rationale for incorporating nutritional care into public health policies and clinical practice. Search criteria included articles published in English within the last 4 years and pertaining to older adults.

**TABLE 1:** Recent studies on nutritional interventions to support healthy aging in older adults.

Author, year, citation, (link), study country/site	Study design	Key results and conclusions
**Nutrition screening/Assessment**		
Bloom et al. (72) (https://doi.org/doi: 10.1007/s40520-022-02171-3) England/Community dwelling	Compares nutrition risk on the DETERMINE Checklist and the MUST tool DETERMINE uses 10 questions on dietary habits and socioeconomic factors that affect food intake. MUST is a score for nutrition risk based on Body Mass Index, unintentional weight loss, and acute illness with reduced food intake.	Among 176 adults (median age of 83.3 years), almost half (47%) had a moderate or high score for nutritional risk using the DETERMINE Checklist, as compared to 8% using MUST. The higher nutrition risk scores from the DETERMINE Checklist were associated with poorer self-reported physical function and higher odds of being frail. Based on the study findings, the DETERMINE Checklist may be of value in screening community-dwelling older adults for early nutritional inadequacy and as a predictor of poor physical function and frailty.
Tey et al. (83) (doi: 10.1038/s41598-021-02274-3), Singapore/Community dwelling	Cross-sectional study to determine the prevalence of low ASMI (ASM/height^2^) and factors associated with low ASMI in older community-dwelling adults	1211 subjects (mean age of 73.2 years and body mass index 20.43 kg/m^2^) participated in this study. Most were Chinese (85.5%) and >94% had a “0” Charlson Comorbidity score. More than half (57%) had vitamin D deficiency or insufficiency. There was a 60% prevalence of low ASMI in the study group with no significant difference between genders (57% males vs. 61.8% females, *P* = 0.1068). In adults at risk for malnutrition, the prevalence of low ASMI was 81.3% and 20.6% in adults with normal nutritional status (*P* < 0.001). Other risk factors included older age, smaller calf circumference, and lower bone mass. Early identification and management of low ASMI in older adults can reduce health complications, support function, improve quality of life, and reduce healthcare costs.
Cheong et al. (102) (doi: 10.3390/nu12113329), Singapore/Community dwelling	Cross-sectional study to identify the levels of nutritional biomarkers in community-dwelling older adults >65 years with normal nutritional status (MUST score of 0) and to determine factors that impact nutritional biomarkers	The study evaluated 400 participants (54.25% women) with mean age of 71.21 ± 0.26 years and mean BMI of 24.53 ± 0.15 kg/m^2^. Most older adults had normal levels of prealbumin, albumin, total protein, creatinine, zinc, corrected calcium, vitamin B12, ferritin and hemoglobin. More than half (52%) had low serum 25(OH)D (<30 mcg/L); 13.5% had vitamin D deficiency, and 38.5% had vitamin D insufficiency. Researchers found low serum zinc levels (<724 mcg/L) in 10% of the older adults. These findings highlight unrecognized nutritional deficiencies in older community-dwelling adults with normal nutritional status. Public health nutrition programs targeting these findings can help identify and treat these nutrient shortfalls and preserve optimal health in community-dwelling older populations.
McKeever et al. (103) (doi: 10.14283/jarcp.2019.2) United States/Community dwelling	Cross-sectional study to examine independent predictors of inadequate dietary intake and poor diet among a multi-ethnic group of urban community dwelling older adults using the DETERMINE checklist to identify those who would benefit from ONS usage	In phone interviews, the DETERMINE Checklist was used to determine nutritional status for *n* = 1001 ethnically diverse participants (69% female), mean age 66.9 years (± 6.4). Most participants were at moderate- or high-risk for malnutrition (78.7%). Domains predicting inadequate dietary intake were social isolation, lower educational levels, food insecurity, altered ADLs, polypharmacy, or having >3 alcoholic drinks per day. Of those who met criteria for nutritional shortfall, <50% of reported consuming ONS in the previous 6 months. Dietary inadequacy is prevalent in community-dwelling older adults, and the use of ONS seems beneficial for preventing and treating nutritional decline.
**Nutrition intervention/Outcomes**		
Chavarro-Carvajal et al. (104) (doi: 10.1016/j.clnesp.2022.01.032), Colombia/Community dwelling)	Nutrition-focused QIP (nutrition education, physical exercise, and use of ONS for 60 days) by community dwelling older adults (>60 years) with malnutrition or at-risk for malnutrition	The nutritional QIP study included 618 participants (69.4% female), mean age of 74.1 ± 8.7 years, with an average of 2.6 comorbidities and medium socioeconomic status (76%) completed the study. Of those, 324 (52.4%) had significant improvement in MNA-SF scores, calf circumference, and maintenance or improvements in body weight and BMI following the nutritional QIP intervention. Community-dwelling, older adults commonly experience malnutrition or its risk when living with chronic illness or following a hospitalization for acute illness. Nutrition education and the use of ONS can improve nutritional status in such individuals.
Gomez et al. (87) (doi: 10.1016/j.clnu.2022.05.003), Colombia/Community dwelling	Nutrition-focused QIP (nutrition education, physical exercise, dietary counseling, and use of ONS for 60 days) by community dwelling adults (>60 years) with malnutrition or at-risk for malnutrition	The study included 618 participants (69.4% female), mean age of 74.1 ± 8.7 years, with an average of 2.6 comorbidities (28.5% cardiovascular and respiratory disease) completed the study. Researchers found significant improvements (*P* < 0.001) in cognition (MMSE), physical function (ADL and SPPB), affective disorder status (GDS), and health-related quality of life (EQ-VAS). Self -reported QOL (EQ-5D-3L) scores were also improved. Nutrition education and use of ONS can maintain and improve mental and physical function, thus supporting quality of life in community dwelling older adults with chronic diseases.
Chew et al. (105) (doi: 10.1016/j.clnu.2020.10.015), Singapore/Community dwelling	Randomized, controlled clinical trial to determine the effects of 2 daily servings of an ONS containing HMB and vitamin D along with dietary counseling in community dwelling adults >65 years at medium or high risk for malnutrition at 30, 90, and 180 days	805 participants (60% female), with mean age of 74.15 and a mean body weight of 45.32 kg and mean BMI of 18.42 kg/m^2^ completed the study. Most participants were Chinese ethnicity (87%). A significantly higher percentage of older adults in the intervention group achieved the primary composite outcome of survival without hospital readmission and with at least 5% weight gain by day 180 compared to placebo (33.4% vs. 8.7%, P < 0.001). Significantly improved nutritional and functional outcomes were achieved in the study group. Nutrition intervention with ONS containing HMB and vitamin D along with dietary counseling reduced malnutrition risk and improved nutritional and functional status in community dwelling older adults.
Smith et al. (106) (doi: 10.3390/nu12020517), England/Community dwelling	Randomized, controlled clinical trial to determine the effects of ONS and dietary advice (DA) on oral intake, weight, QOL, healthcare utilization and satisfaction in malnourished community dwelling older adults	308 participants (67% female) mean age 71.5 ± 10.7 years, with medium (44%) and high risk (56%) for malnutrition (MUST) completed the study. There was significantly greater total energy, protein intake and weight gain in the study group receiving ONS and DA (+401 kcal/d, *P* < 0.001; +15 g/d, *P* < 0.001; +0.8 kg; *P* < 0.001) compared to DA alone. Significant reductions (HCP visits 34%, emergency admissions 50%, LOS 62%) in health care utilization were noted in the ONS/DA group. Nutrition intervention utilizing ONS, and dietary counseling improved oral intake, increased body weight and reduced healthcare utilization in community dwelling malnourished older adults.
Riley et al. (94) (doi: 10.1002/jpen.1606) United States/Community dwelling	Nutrition-focused QIP (admission nutrition screening, patient and caregiver education, and use of ONS) on 90-day hospitalization rates and healthcare utilization by at-risk or malnourished patients admitted to home health care	2,206 (38.8%) participants were identified as at risk for malnutrition with 1,546 (70%) meeting the QIP criteria. QIP patients had a mean age of 76.8 years with 83.5% ≥ 65 years; a majority (67.9%) were admitted to home care after hospital discharge. Results showed significant reductions in relative risk of hospitalization after enrollment in the QIP program—24.3% at 30 days, 22.8% at 60 days, and 18.3% at 90 days when compared to the historic control group, and 18.2, 16.2, and 12.1% when compared to the concurrent group. Total cost savings realized from the reduced healthcare utilization at 90 days were $2,318,894 or $1,500 per patient. For older adults with malnutrition or its risk, implementation of a home-care nutritional QIP (including ONS use) reduced rehospitalization rates and healthcare utilization, leading to improved healthcare outcomes and significant cost savings.
Smith et al. (107) (doi: 10.3390/ijerph17103590) United States/Community dwelling	Community based workshop focused on identifying and managing malnutrition to reduce fall risk in older adults	429 participants attended the SUYN (Stepping Up Your Nutrition workshop) with 38% (*N* = 163) participating in the follow-on Stepping On (SO), an evidence- based fall prevention program. Average age of the SUYN participants was 74.71 (±11.45) with 33.7% older than 80 years. Majority of the participants were female (63.3%). Malnutrition risk was measured in the SUYN participants by SCREEN II with high and moderate malnutrition scores identified in 71% and 20%, respectively. Of those participants at high malnutrition risk, 79.1% attended the SO workshop. Identification of malnutrition risk among community-living older adults is critical to ensuring participation in community-based programs focused on nutrition and fall prevention.
**Clinical practice implications**		
Hong et al. (108) (doi: 10.36648/1479-1072.22.30.01-05) United States/Community dwelling	Assess health care provider (HCP) and patient satisfaction with the nutritional QIP program implemented at 3 healthcare system clinics	Following the implementation and execution of a nutritional QIP at three healthcare clinics, 187 (31.1%) of the patients (>45 years) completed a QIP satisfaction survey. Overall, most patients were very satisfied with the overall nutrition care (81.8%) and nutrition education (81.3%) provided in the QIP program and the healthcare providers’ ability to answer nutrition related questions (82.4%). Higher adherence (*n* = 100, 53.5%) to the prescribed ONS regime was noted when QIP patients were highly satisfied with the nutrition care provided. Healthcare providers reported high levels of confidence and increased satisfaction over time with the use of the QIP program (4.6/5 points). Thus, the use of a nutritional QIP program can drive high levels of patient and HCP satisfaction, improve efficacy of nutritional care, and reduce healthcare costs in the community setting.
Rodriguez-Sanchez et al. (109) (doi: 10.2147/CEOR.S256671) Spain/Community dwelling	Cross-sectional and longitudinal analyses on the impact of malnutrition risk on healthcare utilization and costs in community dwelling older adults	*N* = 1660 older adults (ages 66–98 years) were assessed for nutrition status according to the GLIM criteria. Adults with malnutrition risk were classified according to GLIM phenotypic and etiologic criteria. One out of four (27.5%) older adults living in the community were at risk for or had malnutrition. The presence of malnutrition was associated with higher healthcare utilization and increased costs related to more frequent hospitalizations, longer LOS, higher costs, and polypharmacy. Nutrition screening and counseling along with nutrition intervention can improve health of older community-living adults and lower healthcare costs.
Elia et al. (110) (doi: 10.1016/j.clnu.2015.07.012) United States/Europe/Community dwelling/Care homes	Systematic review (22,819 publications) with meta-analysis to determine if the use of ONS can result in cost savings and drive cost-effective outcomes in adults living in the community and in care homes.	A cost analysis with secondary clinical outcome measures was conducted on data from 19 clinical studies. In 9 studies/economic models using ONS for <3 months, researchers found consistent savings (median cost savings 9.2%, *P* < 0.01). With ONS use for ≥3 months (five studies), the median cost savings was 5% (*P* < 0.05). Meta-analysis revealed that ONS usage reduced hospitalization by 16.5% (*P* < 0.001). ONS use in the community, regardless of use in the hospital, was associated with overall net cost savings or near-neutral balance with clinical outcomes such as reductions in infections, minor post-operative complications, falls and functional limitations, and improved quality of life. Authors concluded ONS use should be an integral part of community clinical practice for patients who were malnourished or at-risk; this intervention reduced complications and improved quality of life while reducing the cost of care.
Sulo et al. (92) (doi: 10.1016/j.vhri.2022.08.005) Colombia/Community dwelling	Economic model for budget-impact and cost-effectiveness analyses The modeling was based on real-world findings from a nutrition QIP study of older, community-living individuals attending outpatient clinics and identified as at-risk/malnourished (*n* = 618) QIP intervention included nutrition screening, dietary education, lifestyle counseling, 60-day consumption of ONS, and 90-day follow-up.	With the QIP nutritional intervention over 90 days, total use of healthcare resources was reduced by >40% (*p* < 0.001). Hospitalizations were lowered by about 80%, ED visits by >60%, and outpatient clinical visits by nearly 40% (*p*-values < 0.001). Based on economic modeling, total cost savings of $129,740 or per-patient cost savings of $210 over 90-days were attributed to the nutritional QIP intervention. Total cost savings equated to nearly twice the initial investment for the intervention, i.e., a per-dollar return on investment of $1.82, a finding that clearly supports “value” in nutrition care.

ADL, activities of daily living; ASMI, low appendicular skeletal muscle mass index; BMI, body mass index; ED, emergency department; EQ-VAS, EuroQoL-visual analog scale; HMB, beta-hydroxy-beta methylbutyrate; LOS, length of stay; MMSE, mini-mental state exam; MNA-SF, mini nutrition assessment short form; MUST, malnutrition universal screening; ONS, oral nutritional supplements; QIP, quality improvement program; SPPB, short physical performance battery; GDS, global depression scale; GLIM, Global Leadership Initiative on Malnutrition; QoL, quality of life.

## 3. Health economic studies show “value” in nutritional care

Beyond tolls to health and wellbeing, poor nutritional status increases use of healthcare resources with concordant rises in costs for healthcare in children (89, 90) and in young and older adults (Table 1). The field of Health Economics and Outcomes Research (HEOR) has emerged over the past decade to measure the link between treatments and actual outcomes, including cost outcomes. HEOR thus provides evidence-based guidance on how to improve care. In HEOR terms, the “value” of a care strategy is determined by outcomes relative to costs; the numerator of a value equation is outcome, while the denominator is cost (91). Nutritional care is advantageous when it improves outcomes. The “value” of nutritional care is recognized when patients’ health outcomes improve at reasonable incremental costs for nutritional care and with reduced overall costs of care (91).

There are many studies to illustrate the “value” of nutritional care. A recent cost-modeling study was conducted among older, community-living adults in Colombia (92). Following a nutrition-focused intervention with ONS use, total healthcare resource needs over a 90-day interval were reduced by more than 40% (hospitalizations ↓80%, emergency department visits ↓60%, and outpatient clinical visits ↓40%; *P* < 0.001). Based on economic modeling, per-patient cost savings of $210 over 90 days was attributed to this nutritional care. Total cost savings was consistent with nearly twice the initial investment for Quality Improvement Program (QIP) intervention; that is, the per-dollar return on investment was $1.82 (92). In a cost study from the USA, use of an advanced enteral nutritional formula in hospitalized patients with sepsis was estimated to save at least $52 million annually (93). Another study of older patients in home healthcare programs showed that rates of hospitalization and healthcare resource use were significantly reduced by prescribing ONS for adults at-risk/malnourished; cost-savings from 90-day healthcare resource utilization was approximately $1500 per patient treated (94). Numerous other studies and reviews underscore cost savings/cost effectiveness of nutritional interventions, especially ONS (95–101).

## 4. Conclusion

Our *Perspective* documents clear links between nutritional sufficiency in early life and better chances of good health, sustained functionality, and maintained wellbeing in older age. We now call on healthcare clinicians and public health professionals to act against poor nutrition in infants, children, and adults. We propose the following specific nutrition-related actions to meet UN and WHO goals of *Zero Malnutrition* and *Good Health and Wellbeing* across the lifespan.

• Build programs worldwide for professional training on nutritional awareness and care.
• Gain consensus among health professionals on the best tools for measurements of nutritional risk and outcomes.
• In some situations, it may be necessary to develop new nutritional screening and assessment tools. It is important to seek tools that result in easy and actionable steps for both the healthcare provider and the patient.
• Train professionals on use of best-practice tools.
• Use outcome studies to inform best-practice nutritional care across the lifespan.
• Create policies to emphasize nutritional care in communities, hospitals, and long-term care facilities worldwide.

## Data availability statement

The data presented in this study are included in this article/Supplementary material, further inquiries can be directed to the corresponding author.

## Author contributions

SS conceived the manuscript concept. SS and CG participated actively in the manuscript drafting. LR-M and RM served as expert consultants/editors on content for the geriatric and pediatric sections, respectively. All authors reviewed, edited, and approved the final version of the manuscript.
