# Supplementary material for: Good nutrition across the lifespan is foundational for healthy aging and sustainable development
Source: Front Nutr. 2023 Jan 24;9:1113060. doi: 10.3389/fnut.2022.1113060 (PMC9902887; doi:10.3389/fnut.2022.1113060)
Supplement: Supplementary file 1 [file Data_Sheet_1.docx]

**Supplemental Materials**

**Supplemental Box 1. Summary of suggested approaches for ending malnutrition and improving health and wellbeing across the lifespan**

**Approaches to end malnutrition and improve health and wellbeing across the lifespan**

- Develop programs and policies to foster development of sustainable food and nutritional programs.
- Integrate effective nutritional tools into practice—tools for screening and assessment and tools for measuring outcome results.
- Build consensus on measures and cutoffs; provide clinician training for use of tools.
- Utilize follow-up monitoring to ensure that nutrition-related policies, practices, and training yield benefits in healthy aging as well as in improved health outcomes and reduced healthcare costs.

**Supplemental Figure 1. Muscle strength and functionality in older age are affected by nutrition across the lifespan.** Adapted from Cruz-Jentoft et al. Age Ageing. 2019;48(1):16-31

**
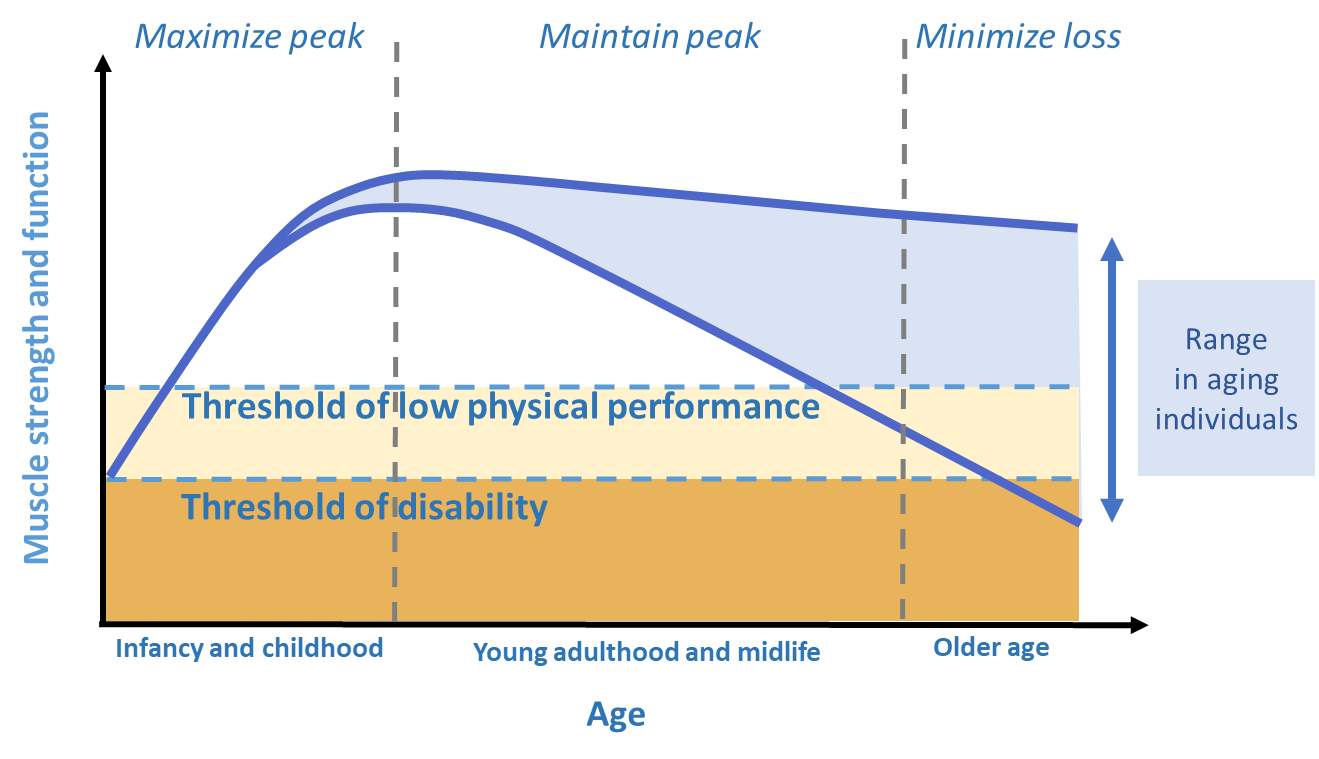
**

**Supplemental Figure 2. Healthy Aging: quantifying the impact of nutritional interventions for older adults**

**
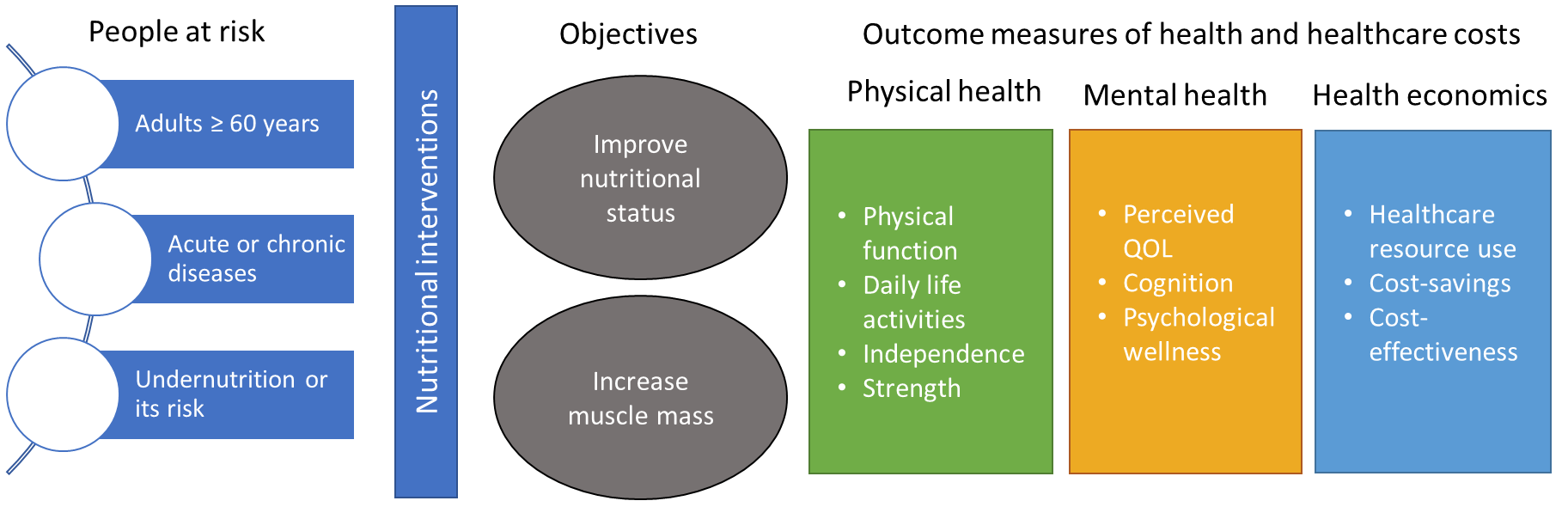
**
